# Supplementary material for: Metabolomic Profiling in Patients with Different Hemodynamic Subtypes of Severe Aortic Valve Stenosis
Source: Biomolecules. 2023 Jan 3;13(1):95. doi: 10.3390/biom13010095 (PMC9855798; doi:10.3390/biom13010095)
Supplement: Supplementary file 1 [file biomolecules-13-00095-s001.zip › biomolecules-2033101-supplementary-finaling/biomolecules-2033101-supplementary.pdf]

Supplementary Material

Metabolomic profiling in patients with different hemodynamic subtypes of severe aortic valve stenosis

Philipp Bengel, Manar Elkenani, Bo E. Beuthner, Maik Pietzner, Belal A. Mohamed, Beatrix Pollok-Kopp, Ralph Krätzner, Karl Toischer, Miriam Puls, Andreas Fischer, Lutz Binder, Gerd Hasenfuß, Moritz Schnelle

Correspondence: philipp.bengel@med.uni-goettingen.de & moritz.schnelle@med.uni-goettingen.de

The file includes:

Supplementary Figure S1: Nitrogen metabolites in AS and healthy controls

Supplementary Figure S2: Amino acids (AAs) metabolites in AS and healthy controls

Supplementary Table S1: Medical history

Supplementary Table S2: Pharmaceutical medication

Supplementary Figure S1.

A

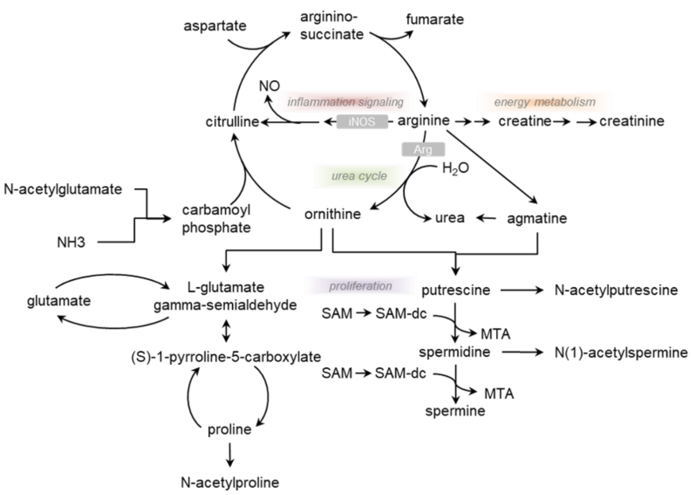

B

Sub Pathway	Biochemical Name	All AS / Control
Urea cycle	arginine	0.78
	urea	1.56
	ornithine	0.88
	3-amino-2-piperidone	1.38
	2-oxoarginine*	0.83
	citrulline	0.97
	homoarginine	0.78
	homocitrulline	2.12
	proline	0.86
	dimethylarginine (SDMA + ADMA)	1.43
	N-acetylarginine	0.8
	N2,N5-diacetylornithine	3.57
	N,N,N-trimethyl-alanylproline betaine (TMAP	2.04
	argininate*	0.97
	dimethylguanidino valeric acid (DMGV)*	3.65
Polyamine Metabolism	N-acetylputrescine	1.63
	N-acetyl-isoputreanine	2.53
	spermidine	1.22
	(N(1) + N(8))-acetylspermidine	2.29
	acisoga	2.35
	spermine	1.47
	5-methylthioadenosine (MTA)	1.75
	4-acetamidobutanoate	2.56

C

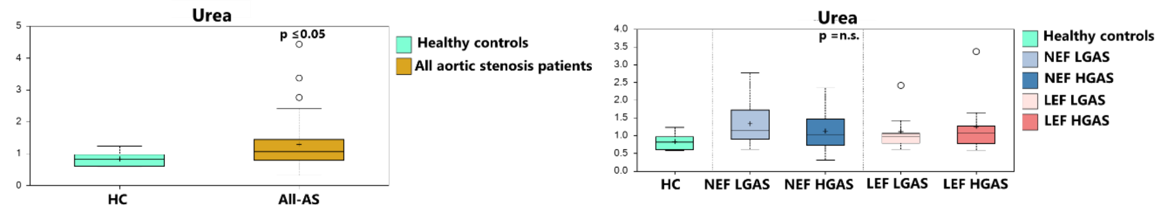

Figure S1. Nitrogen metabolites in AS and healthy controls. A: Schematic depicting the urea cycle and polyamine metabolic pathways. **B:** list of altered metabolites. Significantly up- and downregulated molecules are marked in red and green, respectively. Values marked in light green indicate a trend towards statistical significance (0.05< p <0.10). Non-colored values are not significantly different for that comparison. Numbers indicate x-fold increase/decrease in concentration. **C:** Urea as an example between All-AS vs controls as well as the different AS subgroups. On each box, the black cross indicates the mean value, the borders and segmentation indicate limits of upper / lower quartile and median values. Outliers are plotted as circles. n.s.: not significant between AS subgroups.

Supplementary Figure S2.

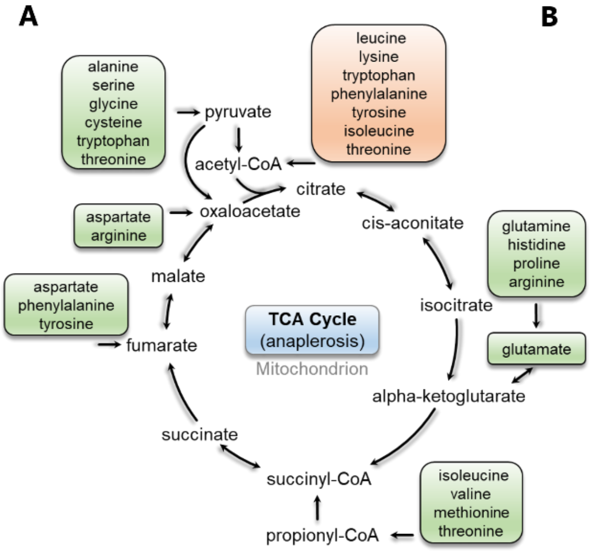

B

Sub Pathway	Biochemical Name	All AS / Control
Amino Acids	arginine	0.78
	aspartate	0.95
	glycine	0.78
	histidine	0.67
	isoleucine	0.85
	leucine	0.8
	lysine	0.84
	methionine	0.76
	phenylalanine	0.99
	proline	0.86
	serine	0.88
	glutamate	1.07
	glutamine	0.99
	threonine	0.69
	tryptophan	0.77
	valine	0.86
	cysteine	1.06
	alanine	0.81
	tyrosine	0.82
	asparagine	0.84

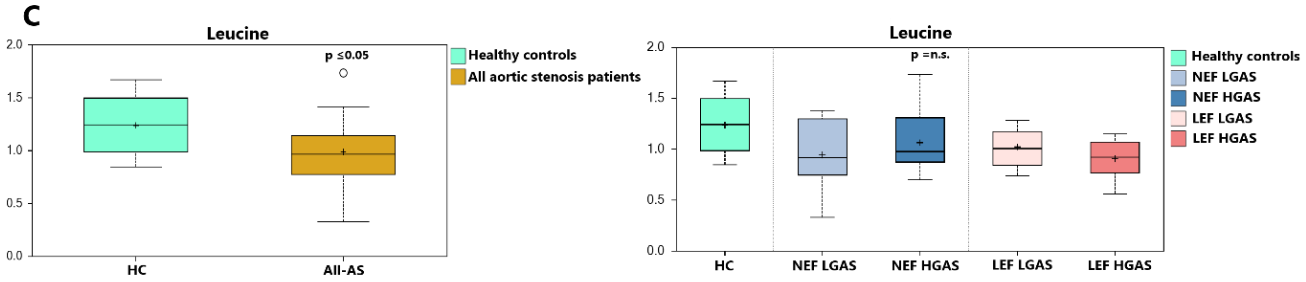

Figure S2. Amino acids (AAs) metabolites in AS and healthy controls. A: Illustration of AAs utilization pathways for energy production via TCA-cycle. **B:** list of altered metabolites. Significantly downregulated molecules are marked in green. Value marked in light green indicates a trend towards statistical significance (0.05< p <0.10). Non-colored values are not significantly different for that comparison. Numbers indicate x-fold decrease in concentration. **C:** Leucine as an example between All-AS vs controls as well as the different AS subgroups. On each box, the black cross indicates the mean value the borders and segmentation indicate limits of upper / lower quartile and median values. Outliers are plotted as circles. n.s.: not significant between AS subgroups.

Supplementary Table S1:

Table S1. Medical history.

	NEF HGAS	LEF HGAS	LEF LGAS	NEF LGAS
**Medical history, n (%)**				
Coronary artery disease	4 (40%)	5 (50%)	9 (90%)	7 (70%)
Prior myocardial infarction	0 (0%)	0 (0%)	3 (30%)	1 (10%)
Prior Stroke / TIA	1 (10%)	0 (0%)	1 (10%)	2 (20%)
Peripheral artery disease	0 (0%)	1 (10%)	2 (20%)	0 (0%)
Hypertension	10 (100%)	5 (50%)	9 (90%)	10 (100%)
Atrial fibrillation	1 (10%)	3 (30%)	6 (60%)	7 (70%)
Chronic kidney disease (GFR <60 ml/min/1,73m^2)	7 (70%)	2 (20%)	6 (60%)	8 (80%)
Hyperlipidemia	8 (80%)	3 (30%)	3 (30%)	5 (50%)
Diabetes mellitus	4 (40%)	3 (30%)	5 (50%)	5 (50%)

Supplementary Table S2:

Table S2. Pharmaceutical medication. ACE-Inhibitor: Angiotensin-converting enzyme inhibitors, AT1-Blocker: Angiotensin II receptor type 1 blockers, ARNI: Angiotensin receptor II blocker - neprilysin inhibitor, MRA: Mineralocorticoid receptor antagonists, VKA: Vitamin K antagonists, DOAC: Direct oral anticoagulants.

	NEF HGAS	LEF HGAS	LEF LGAS	NEF LGAS
**Medication, n (%)**				
ACE-Inhibitor	6 (60%)	4 (40%)	6 (60%)	6 (60%)
AT1-Blocker	3 (30%)	2 (20%)	3 (30%)	3 (30%)
ARNI	0 (0%)	1 (10%)	0 (0%)	0 (0%)
Beta-Blocker	7 (70%)	5 (50%)	5 (50%)	6 (60%)
MRA	2 (20%)	3 (30%)	1 (10%)	0 (0%)
Loop Diuretics	4 (40%)	7 (70%)	7 (70%)	7 (70%)
Thiazid Diuretics	4 (40%)	2 (20%)	2 (20%)	4 (40%)
Statines	5 (50%)	4 (40%)	3 (30%)	6 (60%)
Ezetemibe	0 (0%)	0 (0%)	1 (10%)	0 (0%)
VKA	0 (0%)	2 (20%)	2 (20%)	3 (30%)
DOAC	1 (10%)	1 (10%)	1 (10%)	4 (40%)
Insulin	0 (0%)	1 (10%)	2 (20%)	3 (30%)
Oral Antidiabetics	4 (40%)	2 (20%)	2 (20%)	2 (20%)
